# Acral lentiginous melanoma with multiple bone metastasis: case report

**DOI:** 10.11604/pamj.2023.45.141.40508

**Published:** 2023-07-24

**Authors:** Pei Ting Chen, Kapilkumar Manvar, Rashid Chaudhry, Richard Wu, Jen Chin Wang

**Affiliations:** 1 Division of Hematology/Oncology, Brookdale University Hospital Medical Center, Brooklyn, New York, USA

**Keywords:** Acral lentiginous melanoma, cutaneous melanoma, bone metastasis, case report

## Abstract

Acral lentiginous melanoma (ALM) is a type of melanoma that is traditionally seen on the soles of the feet, palms of the hand, and under the fingernails or toenails. It is the least frequently diagnosed melanoma among the four histologic subtypes of cutaneous melanoma, accounting for less than 5% of all cases. ALM is frequently diagnosed at late stages and has higher incidences in non-white populations in relation to the other forms of cutaneous malignant melanoma. The most common sites of metastases in melanoma are the skin and subcutaneous tissue followed by lung, liver, brain, and bone. Bone metastases from malignant melanoma usually occur in patients who already have widespread metastases. We present this paper as a unique case study of ALM lesion in an 84-year-old African American male presenting originally in the base of right fifth toe plantar aspect then found multiple bone metastases without any other organ involved.

## Introduction

Melanoma can be divided into four main subtypes based on the growth pattern: superficial spreading melanoma (70%), nodular melanoma (5%), lentigo maligna melanoma (4-15%), and acral lentiginous melanoma (2-3%). Acral lentiginous melanoma is a rare subtype of melanoma arising on the hands and feet (palms, soles, fingers, toes, and nail units) [1]. Melanoma can spread to the subcutaneous tissue that is underneath the skin, the lymph nodes, lungs, liver, brain, bone and other organs throughout the lymphatic system and/or the blood vessels. Bone is the fourth frequent distant metastatic organ. Melanoma bone metastasis usually occur in patients who already have widespread metastases in other organs [2]. Herein, we present a rare case of ALM lesion which was originally found in the base of right fifth toe plantar with multiple bone metastases but without any other organs involving.

## Patient and observation

**Patient information:** an 84-year-old African American male patient with past medical history of prostate cancer status post external beam radiation therapy 6-7 years ago, hypertension, hyperlipidemia and inability to ambulate for past four days was admitted due to complaints of mild back radiculopathy pain radiating around upper abdomen in a belt-like fashion for three months, worsening constipation, and abdominal discomfort.

**Figure 1:**
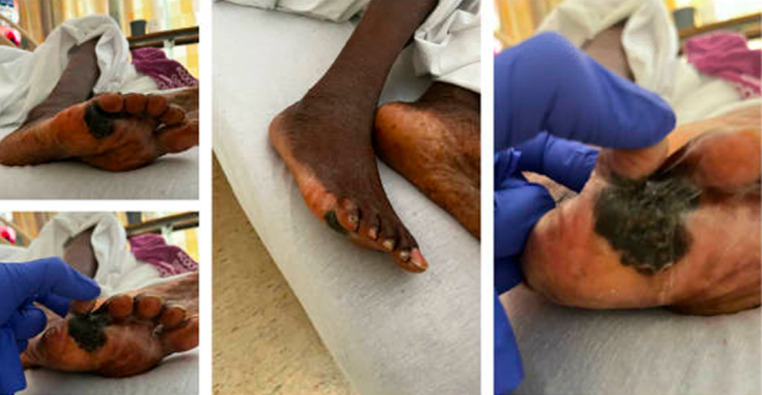
well demarcating irregular hyperpigmenting patch seen at the base of the right fifth toe plantar aspect which extends to the space between the 4^th^ and 5^th^ toes

**Clinical findings:** on physical examination, the patient presented with mild facial weakness on left with tongue deviation to the right, left arm drift and mild paraparesis worse on right side. Hip flexion and knee flexion were graded at muscle strength 2/5 with other muscle strengths of 4/5. There is a well demarcated irregular hyperpigmented patch seen at the base of right fifth toe plantar aspect which extends to the space between 4^th^ and 5^th^ toes (Figure 1), with no lymphadenopathy or splenomegaly noted.

**Timeline:** the patient reported the skin lesion being present for the last two or three years; the lesion being asymptomatic with no itchiness or pain.

**Figure 2:**
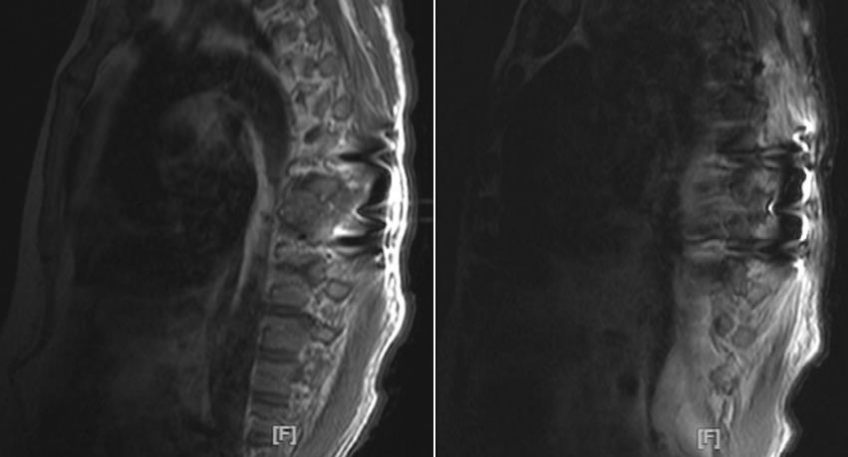
large lesions seen in the T8 vertebra with epidural extension of disease and spinal cord compression

**Figure 3:**
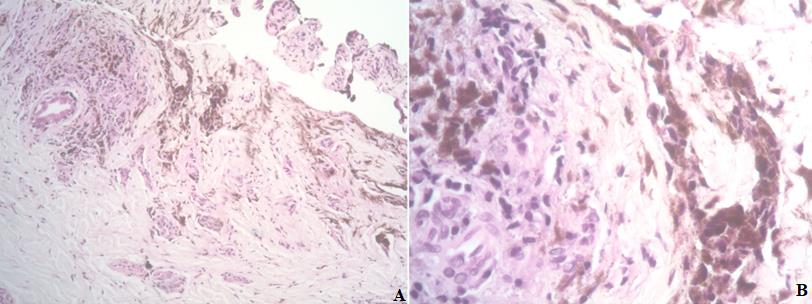
melanoma at right foot; A) at intermedium magnification (100 x), the atypical melanocytes infiltrate asymmetrically in the ulcerated upper dermal tissue, the melanin incontinence is present; B) at high magnification (400 x), the invasive components of oval to spindle atypical cells admixed with macrophages

**Figure 4:**
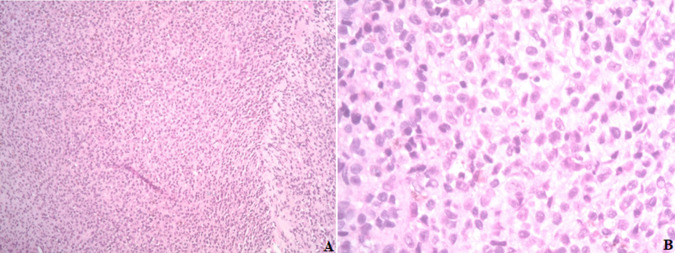
metastatic melanoma in spine; A) at intermedium magnification (100 x), the sheets of atypical melanocytes present in the spinal tissue; B) at high magnification (400 x), the majorities of the atypical melanocytes are oval and spindle cells with vesicular nuclei

**Figure 5:**
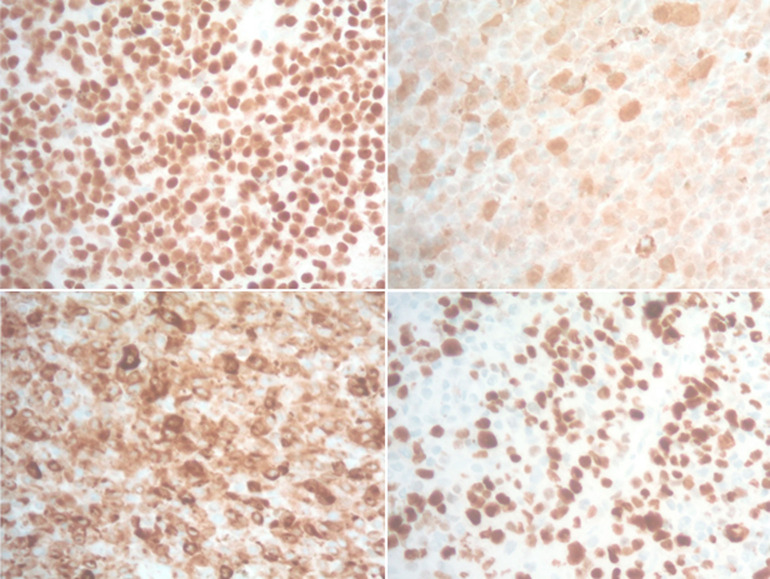
the metastatic melanoma in spine; the neoplastic cells diffusely and strongly express the Sox10, S-100 and melanin, which confirms the diagnosis of metastatic malignant melanoma; the metastatic melanoma in spine; the melanoma demonstrates 60-70% proliferative rate

**Diagnostic assessment:** initial lab results were insignificant, with normal liver and renal function of PSA less than 0.1. A computed tomography scan without contrast of the abdomen and pelvis revealed no acute abdominopelvic pathology but noted a near complete lytic replacement of the visualized T8 vertebral body, right iliac sclerotic foci, and right iliac wing lytic lesion. Patient's facial asymmetry, tongue deviation, and left arm drift recovered later during stay, but the bilateral lower extremity weakness worsened. Dermatology consult suspected acral melanoma versus acral melanocytic nevus and had biopsy of the skin lesion on right foot. Neurology was consulted for the bilateral lower extremity weakness and lower back pain with suspected partial spinal cord compression at T8, also has evidence of cerebral dysfunction. Then MRI of the brain and whole spine showed extensive findings of calvarial bone metastatic disease and no brain metastasis. Large bone lesions were seen in the T8 vertebra with epidural extension of disease and spinal cord compression, and multilevel degenerative changes on lumbar spine area (Figure 2). Patient had T8 laminectomy and resection of dorsal epidural tumor and T7-9 posterior fusion through neurosurgery. The biopsy of the right plantar foot lesion showed segment of dermis containing atypical melanocytic proliferation (Figure 3). Thoracic spinal epidural tumor showed sheets of atypical melanocytes present in the spinal tissue (Figure 4, A & B). A. At intermedium magnification (100 x), the sheets of atypical melanocytes present in the spinal tissue. B. At high magnification (400 x), The majorities of the atypical melanocytes are oval and spindle cells with vesicular nuclei. The tumor cells were immunoreactive to Sox-10, S-100, melanA, HMB-45, CD56 and not immunoreactive to AE1/AE3, CAM5.2, Desmin, GFAP, and SYN which is consistent with malignant melanoma, the ki-67 was 60-70% (Figure 5). The Ct DNA (liquid biopsy): NTRK1 A635T 1% VUS with next gen sequencing was negative for actionable mutation.

**Therapeutic intervention:** patient was diagnosed with metastatic acral melanoma and was started on doublet ICT (immune checkpoint therapy- Nivolumab+Ipilimumab) with radiation therapy. The patient received steroid therapy which showed improvement of bilateral lower extremity weakness.

**Follow-up and outcomes:** after two cycle of immune checkpoint therapy, the patient developed severe hypercalcemia (calcium level of 14 mg/dL) which were reported possible related to the ipilimumab administration. Patient was treated with intravenous fluid, calcitonin and zoledronic acid and steroid. But then follow up communication was lost with the patient after discharge as he moved back to his hometown in Jamaica.

**Ethics approval:** ethics approval to report this case was obtained from Brookdale Hospital Institutional Review Board. Our institution does not require ethical approval for reporting individual case reports.

**Informed consent:** informed written consent was obtained.

## Discussion

Melanoma, which refers to malignancy of melanocytes, is divided into four subtypes: superficial spreading melanoma, nodular melanoma, lentigo maligna melanoma, and acral lentiginous melanoma. Superficial spreading melanoma, the most common type of melanoma (70%), is related to intermittent sun exposure and hence is often localized in sun-exposure areas such as the legs of women and the back and shoulders of men [1].

Nodular melanoma, which accounts for 5% of melanomas, usually occurs on the trunk and limbs of patients within the fifth or sixth decade of life. Nodular melanoma is more common in males than females. It is often ulcerated with the tendency of more vertical growth than traditional radial growth [2]. Lentigo maligna melanoma correlates with long-term sun exposure and increasing age that may develop for decades before invading the papillary dermis. It accounts for 4%-15% of all melanoma patients [3]. Acral lentiginous melanoma, the rarest type of melanoma, accounts for 2% to 3% of cases. It arises on non-sun-exposed areas such as glabrous skin and adjacent skin of digits, palms, soles, or nail beds of the great toe or thumb. It commonly occurs in darker-skinned populations such as Asians, Hispanics, and African American [1]. Among all the four subtypes of melanoma, ALM has the worse prognosis and an inferior survival rate according to several studies [3]. However, it is still unclear whether the reason for the poor prognosis is due to the advanced stage at presentation and delay in diagnosis or the biological aggressiveness of the subtype [4].

Melanoma can spread and affect all organs based on clinical and radiologic findings. The most common site of metastases in melanoma is skin (13%-38%), followed by lung (18%-36%), distant lymph nodes, (5%-34%), liver (14%-20%), brain (2%-20%), bone (4%-17%), adrenal gland (1%-11%), gastrointestinal tract (1%-8%), pleura (3%), pancreas (3%), heart (<1%), kidneys (<1%), thyroid gland (<1%), and uterus (<1%) [4]. Bone metastasis of melanoma usually occurs in patients at later stage with widespread metastases in other organs. Melanoma bone metastases appear as lytic lesions in imaging studies and usually present in the axial skeleton, primarily the skull, vertebral column, pelvis, and ribs. Our case is consistent with melanoma with bone metastasis based on the MRI imaging findings. The MRI imaging changes in this case were limited to the bone including skull and T8 vertebra without brain involvements. The median survival rate of patients with bone metastases from melanoma ranges from 4-6 months and 1-year survival rate is 10%, which indicated the bone metastases are late stage in the progression of melanoma [2].

Mutation of BRAF gene is the most frequently observed mutation in malignant melanoma, accounting for 40%-60% of all cases. However, BRAF mutations are more common in melanoma in regions of high sun exposure. There is 50%-65% of BRAF mutation in superficial spreading melanoma while only 15%-20% in ALM which may explain the lack of high efficacy in BRAF inhibitors in treating ALM [5]. On the other hand, c-KIT mutation and/or amplification are more commonly found in ALM than other types of melanomas which showed promising result of KIT inhibitors such as imatinib in treating patients with KIT mutant melanoma [6]. However, 45-58% of ALM cases are without the KIT or BRAF mutations which were called as triple wild-type, just like our patient, was with limited treatment options [7].

Cytotoxic T-lymphocyte-associated antigen 4 (CTLA-4) and programmed death 1 (PD-1) has revolutionized immunotherapy especially in the treatment of malignant melanoma. Both served as negative regulators of T-cell immune function. In a study of Klemen *et al*. immunohistochemical staining for PD-L1 was performed in all the specimens from patients with melanoma and PD-L1was found expressed in 31% of ALMs cases [8]. Many studies showed long survival in patients with metastatic acral melanoma treated when treated with a combination of anti-CTLA-4 and anti-PD-1. (8) Immune stimulators such as imiquimod, dacarbazine, and interferon may help increase CD8+ tumor-infiltrating lymphocytes within in ALM and thus increase the efficacy of the immune checkpoint inhibitors such as PD-1/PD-L1 inhibitor [5]. Furthermore, lymphocyte activation gene-3 (LAG-3) inhibitor as another checkpoint inhibitor that cause exhaustion of T cell has been found its efficacy in treating advance melanoma [9]. The combination of nivolumab plus relatlimab showed grater benefit with progression-free survival compared to inhibition of PD-1 alone (10.1 months vs 4.6 months) in treating unresectable or metastatic melanoma [10]. Future studies will be needed to assess the efficacy of this combination in treating ALM.

## Conclusion

Acral lentiginous melanoma is a rare subtype of malignant melanoma that is usually found on the soles of the feet, palms of the hand, and under the fingernails or toenails. Compared to other types of melanoma, ALM is more common in non-white population. Bone metastases traditionally present at a late stage of ALM, however our patients were presented with local lesion on plantar with multiple bone metastases. We present this case with the aim to increase the awareness of this rarer type of melanoma which may lead to a better diagnosis and treatment in treating acral lentigo melanoma.
